# A high-quality telomere-to-telomere lumpy skin disease virus genome assembly

**DOI:** 10.1099/jgv.0.002348

**Published:** 2026-10-14

**Authors:** Caroline Wright, Noemi Polo, Sarwar Azam, Graham Freimanis, G. Taru Sharma, Bryan Charleston, Carrie Batten, Tim Downing, Jonas Albarnaz

**Affiliations:** 1 The Pirbright Institute, Surrey, UK; 2 National Institute of Animal Biotechnology (NIAB), Hyderabad, India; 3 Indian Veterinary Research Institute (IVRI), Izatnagar, India

**Keywords:** genome, hybrid assembly, lumpy skin disease, lumpy skin disease virus (LSDV), poxvirus

## Abstract

Lumpy skin disease virus (LSDV) is an emerging livestock capripoxvirus (CaPV) that continues to cause substantial economic losses across Africa, Asia and Europe. However, important uncertainties remain regarding LSDV genome structure, particularly at the telomeric inverted terminal repeats (ITRs) that are central to host interaction, replication and adaptive evolution. Structural variation at the ITRs is driven by recombination during virus replication. Previous assemblies have relied primarily on short-read sequencing, which provides limited resolution of repeat-rich telomeric regions and propagates structural ambiguities into downstream annotation and comparative analyses. Here, we present a high-quality telomere-to-telomere assembly of the LSDV Oman 2009 isolate, generated using a hybrid approach that integrates short Illumina reads with higher-accuracy long Nanopore reads. The resulting 151,091 bp genome contains 157 annotated ORFs and fully resolves complex repeat-rich structures at both ITRs. This new reference corrects misassemblies present in earlier LSDV genomes and confirms clade-specific gene truncations. This genome provides a robust foundation for improved genomic surveillance, accurate read mapping, mutation detection and evolutionary inference of LSDV and demonstrates the value of long-read approaches for resolving complex CaPV genome structures.

## Data Summary

This study used a hybrid short- and long-read sequencing strategy to generate an annotated reference genome for the lumpy skin disease virus (LSDV) Oman 2009 isolate, resolving the architecture of the inverted terminal repeats (ITRs) more accurately than previously available assemblies. The results provide a framework for future hybrid genome assemblies of capripoxviruses (CaPVs) and demonstrate the effectiveness of ONT R10 long-read sequencing for producing high-quality CaPV genomes. Consistent with observations in bacterial systems [1], our findings suggest that long-read data alone may be sufficient for accurate CaPV genome assembly, while short-read data are more effectively applied to resequencing and read mapping [2]. The improved resolution of ITRs and truncated genes in the Oman 2009 genome supports its use as a reliable LSDV reference and facilitates more accurate clade-specific analyses and diagnostic assay development [3]. Continued improvements in sequencing technologies, including ultra-long reads, alternative long-read platforms and improved polymerase accuracy, are likely to enable further refinement of LSDV and CaPV genome assemblies.

## Data Availability

The Illumina and ONT reads are on the SRA under the BioProject accession PRJNA1366067. The ONT reads are available under the accession SRX31142208. The Illumina reads are available under the accession SRX31141470. The genome assembly file and its annotation files are on the Nucleotide database under the accession PV877838. The code used to produce these analyses is publicly available at https://github.com/downingtim/LSDV_hybrid_assembly/. The vaccinia virus entry fusion complex cryogenic electron microscopy structure is available on the RCSB Protein Data Bank under the accession PDB 9UZO.

## Introduction

Lumpy skin disease virus (LSDV, *Capripoxvirus lumpypox*) is an endemic livestock disease in Africa and an emerging threat to bovine health, predominantly in Asia and southern Europe. Owing to its substantial economic impact and rapid transboundary spread, lumpy skin disease is a notifiable disease by the World Organization for Animal Health (WOAH) [4]. Although genomic variation clearly influences LSDV virulence and transmission dynamics, large gaps remain in our understanding of its genome structure, particularly within the highly repetitive terminal regions. These limitations can now be addressed using new technological developments in long-read sequencing approaches.

LSDV is a dsDNA virus that belongs to the *Capripoxvirus* (CaPV) genus of the *Poxviridae* family and is typically classified into three main clades (1.1, 1.2 and 2), with clade 2 representing a recombinant lineage derived from ancestors of clades 1.1 and 1.2 [5, 6]. Currently, circulating field strains are predominantly associated with clade 1.1, whereas clade 1.2 is linked to live attenuated vaccine strains. Most existing LSDV genomes are assembled solely from short-read data, leading to fragmented contigs, incomplete annotation and uncertainty at key loci with high structural complexity. This is especially true for the genes at the terminal 5′ and 3′ ends, e.g. LSDV001 and LSDV156, which are important for host immune modulation [7]. Short-read assemblies may obscure true biological variation in these regions and introduce artefacts that confound phylogenetic interpretation.

The linear 151 kb LSDV genome has a structure typical of poxviruses. It contains 156 annotated ORFs and is flanked by homologous inverted terminal repeats (ITRs) that harbour four ORFs each (ORFs 1–4 and 153–156, respectively) [8] and contain regulatory sequences essential during virus replication. Poxvirus genomes have the two DNA strands covalently linked at the termini, forming an incompletely base-paired hairpin analogous to telomeres at the end of each ITR. Poxviruses replicate their genomes through head-to-head and tail-to-tail concatemeric intermediates, and the conserved sequence that is necessary for concatemer resolution is localized between the hairpins and a series of short tandem repeats [9, 10]. The ITRs and the adjacent 5′ and 3′ regions spanning ORFs 5–23 and 124–152 are among the most variable and encode functions associated with virulence and immune modulation, whereas the central region is highly conserved and encodes genes involved in transcription, DNA replication and virion assembly. Despite their biological importance, the structure of LSDV ITRs remains poorly defined. By contrast, the ITRs of other poxviruses, such as vaccinia virus [9], have been resolved in detail, including long A/T-rich repeats, suggesting a potentially analogous but uncharacterized architecture in LSDV.

Here, we generate a telomere-to-telomere (T2T) annotated reference genome for the Oman 2009 isolate of LSDV by integrating short Illumina reads with long Oxford Nanopore Technology (ONT) reads. This approach used these long reads as the main scaffold for the resulting assembly. It provided an enhanced resolution of the genome, particularly at the repetitive terminal regions, enabling the resolution of complex ITR structures that were previously inaccessible. The resulting high-quality genome provides an improved reference for accurate read mapping, comparative genomics and evolutionary analyses of LSDV and as a template for future CaPV hybrid assemblies.

## Methods

### Sample collection

The LSDV/OMAN/2009/MDBKP2 isolate (‘Oman 2009’) was obtained from the Non-Vesicular Reference Laboratory (NVRL) at The Pirbright Institute. The virus was isolated from a cattle skin nodule biopsy after passaging twice in Madin-Darby bovine kidney cells confirmed free of bovine viral diarrhoeal virus by quantitative reverse transcription PCR, following WOAH protocol guidelines (WOAH Terrestrial Manual 2024).

### Read library preparation and sequencing

For short-read Illumina sequencing, total DNA was extracted from 100 µl of cell culture lysate using a MagMax CORE nucleic acid purification kit and a KingFisher Flex automated extraction robot (Thermo Fisher Scientific) according to the manufacturer’s instructions. The extracted DNA was tagmented and indexed using an Illumina DNA Prep with Enrichment kit (Illumina), with 11 cycles of amplification used during DNA tagmentation. The indexed library underwent targeted enrichment with a custom myBaits panel (Daicel Arbor Biosciences), also used in Breman *et al*. [11] (sequences available as supplementary material). The manufacturer’s instructions were followed for the High Sensitivity Protocol, which included two rounds of probe hybridization at 63 °C. The final library size distribution was determined using a D1000 High Sensitivity ScreenTape on a TapeStation 4200 (Agilent) and quantitated using the Qubit HS kit (Thermo Fisher Scientific). The library was then diluted and re-quantified using the NEBNext Library Quant Kit for Illumina (New England Biolabs) assay. The diluted library was loaded onto an Illumina NextSeq 2000 at a final concentration of 1.6 pM for paired-end sequencing (150 cycles) with a 2% PhiX spike-in.

For long-read ONT sequencing, library preparation was performed using the ONT ligation by sequencing kit (SQK-LSK114) and an input of 340 ng genomic DNA. Final library size distribution was determined using the D1000 genomic DNA ScreenTape on a TapeStation 4200 (Agilent) and quantified using the Qubit DNA BR kit (Thermo Fisher Scientific). Thirty femtomoles of the library were loaded onto a MinION R10.4.1 (FLO-MIN114) flow cell and sequenced for 42 h on a MinION Mk1B device. Basecalling was performed using Dorado 7.3.11.

### Read library quality control and host read removal

Adapter trimming, removal of both short and long reads with low base quality (BQ) scores (phred score <30), exclusion of ambiguous (N) bases, mismatched base pair correction in overlapping regions and cutting of poly-G tracts at 3′ ends were completed using Fastp v0.23.4 (12). Low-quality ONT bases were removed with Nanoq including trimming the 5′ 11 and 3′ 12 bases from all reads [13]. Low-quality Illumina bases with base qualities <30 and reads with lengths <110 bp were removed using the FASTX-Toolkit v0.0.13 (http://hannonlab.cshl.edu/fastx_toolkit/). FastQC v0.12.1 (www.bioinformatics.babraham.ac.uk/projects/fastqc/) was used to verify the effectiveness of Fastp, the FASTX-Toolkit and Nanoq. We estimated species-level abundances for each read using the k-mer classifier Kraken2 (14).

This assigned the reads to the major taxa detected: cattle, LSDV, deer, sheep and human (Table 1). Other taxa were rare (<0.01%). For the ONT data, most reads were unambiguously bovine (72.2%). Across all taxa, the initial ONT read library lengths ranged 35–82,438 bp (median 557 bp) (Table 1). The LSDV-assigned ONT reads were examined further to avoid potential genome assembly errors stemming from host read inclusion. These 209,203 LSDV reads had lengths 35–80,952 bp (median 533 bp) (Fig. S2, available in the online Supplementary Material). This corresponded to 1.9% of the post-quality control reads (187.06 Mbp of data), reflecting the absence of ONT library target enrichment (Table 1). This generated a mean genome-wide read depth of 953-fold when mapped to the final assembly with a mean BQ of 34.3. The paired-end Illumina data was 99% LSDV because probes were used to enrich that library for LSDV (Table 1). This resulted in 39.3 million 151 bp LSDV reads (Fig. S2), resulting in a mean genome-wide read depth of 10,627-fold with a mean BQ of 39.3.

**Table 1.** The numbers of reads at each stage of QC for the ONT and Illumina read libraries, along with the numbers and percentages of reads classified across organisms by Kraken2 The Illumina reads were enriched for LSDV prior to sequencing.

Technology	Initial	Post-Fastp	Post-Fastx	Taxonomic assignment	No. of reads	Percentage of reads
ONT	11,719,320	11,119,320	10,575,827	Cow	7,632,866	72.2
				LSDV	202,760	1.9
				Deer	1,940,662	18.3
				Sheep	623,899	5.9
				Human	175,640	0.2
Illumina	39,918,632	39,818,632	39,718,632	Cow	100,795	0.3
				LSDV	39,323,196	99.0
				Deer	98,340	0.2
				Sheep	98,009	0.2
				Human	98,292	0.2

### *De novo* genome assembly and evaluation

Flye v2.9.1-b1784 was used to assemble the genome from the ONT reads [15]. This used a subset of the longest reads for initial disjointig assembly, ten polishing iterations, an expected genome size of 151 kb and high-quality ONT reads. No Flye polishing improvements were observed beyond four iterations. We created assemblies with ONT reads subject to a size requirement of >500, >1,000, >2,000 and >4,000 bp that either had no effect or assembled less well. Additionally, reducing the coverage for the initial disjointig assembly was attempted but did not improve assembly quality. Assemblies with alternative contigs were excessively long and impaired overall quality. SPAdes v3.13.1 [16] was run in careful mode using a range of input: ONT reads, Illumina reads, both read sets and the same combinations again using contigs from the optimal Flye assembly. We also used Unicycler v0.4.8 [17] to create Illumina-only, ONT-only and hybrid assemblies using conservative, normal and bold modes.

Assembly quality was measured with Quast based on the assembly length, number of contigs, largest contig size, GC content, N50 and number of N bases. To explore local assembly quality, read pileups and breakpoints, we used Minimap2 v2.24-r1122 [18] to index the assembly and map the reads to it. The resulting Sequence Alignment Map files were processed with SAMtools v1.19.2 [19] and SeqKit2 [20] to process, sort and estimate read depth per site. The Binary Alignment Map files stemming from this were examined with IGV v2.16.0 [21] for read alignment and paired-end read consistency inspection. Read-depth variation was visualized using R v4.3.2 [22] packages ggplot2 v3.5.1 [23] and gridExtra v2.3 [24].

### Post-assembly polishing and genome annotation

The assembly was polished using four steps with Medaka, Pilon, Polypolish and Pypolca. Firstly, Medaka v2.0.1 (https://github.com/nanoporetech/medaka) was used to polish the optimal Flye assembly to correct large indel and homopolymer errors using the cleaned ONT reads with a r1041_e82_400bps_sup_v5.0.0 model. The assemblies were compared using Nucmer v3.1 [25]. Secondly, we used Pilon v1.24 [26] to fix single nucleotide polymorphisms (SNPs), indels, gaps, local misassemblies and ambiguous bases over five iterations using the assembly resulting from Medaka. Thirdly, Polypolish v0.6.0 corrected errors in repeats in the Pilon output [27]. To run Polypolish, BWA v0.7.17 mapped the Illumina reads to all possible matches where forward and reverse reads were mapped separately. These reads were filtered by insert size and used for polishing. Fourthly, Pypolca v0.3.1 in careful mode was used to do a final correction step on the Polypolish assembly using the Illumina reads [28]. Canu [29] was used to error-correct and assemble the ONT reads, but this provided no improvement.

The assembly versions were annotated using Prokka v1.14.6 [30] with the --kingdom Viruses option specified, with gene annotation guided by the reference genome KX894508 (155920/2012; 156 ORFs). Annotation was also mapped from the reference strain LSDV NI-2490 (AF325528.1) using Genome Annotation Transfer Utility (GATU) [31]. The annotation was manually inspected by visualizing long- and short-read alignments in IGV v2.16.0 [21] to ensure the gene boundaries were correct. Genome completeness and quality, including ITRs within the terminal regions, were further assessed using CheckV v1.0.3 [32].

### Structural predictions and analyses

Hairpin DNA secondary structure was modelled using the Mfold webserver with default settings (33; http://www.unafold.org). The lowest energy model was visualized with forna [34] (http://rna.tbi.univie.ac.at/forna/).

Protein structure was predicted using a web-based version of ColabFold version 1.6.2 [35] using the predicted amino acid sequence of LSDV042.5 as input, with the predicted aligned error (PAE) displayed with PAE Viewer (36; https://pae-viewer.uni-goettingen.de/). Superimposition of LSDV042.5 model on the structure of the vaccinia virus entry fusion complex (EFC) (PDB 9UZO) [37] and molecular graphics were generated using an open-source build of PyMOL (Schrödinger).

## Results and discussion

### A more accurate LSDV reference genome

We used a combination of long-read ONT and short-read Illumina sequencing data to *de novo* assemble a new reference genome sequence for the LSDV Oman 2009 isolate. The final assembly comprised a single contig of 151,091 bp (N50 151,091 bp), with 157 predicted ORFs and a mean GC content of 25.9% (median 25.6%, Fig. S1). This contig was generated from long reads using Flye v2.9.1-b1784 [15] because Flye has a low chance of making large-scale errors [38]. It was refined through iterative polishing with Medaka v2.0.1 that made 15 changes, Pilon v1.24 [26] that fixed 2 SNP errors and correction with Polypolish v0.6.0 [28] that fixed 9 indel and 2 substitution errors. There were no homopolymer corrections. Alternative assembly strategies resulted in fragmented assemblies with inflated total lengths and numbers of contigs. Overall, polishing with Illumina reads corrected only 28 bp (0.019%) of the 151,091 bp genome assembled from long ONT reads.

The genomic termini of the Oman 2009 reference genome were verified using a known 123 bp hairpin sequence (MN072625.1 [5], which had T-A base differences at bases 30 and 33. The terminal 23 bp of the hairpin contained concatemer resolution sequences (CRSs), which mediate site-specific genome resolution during viral replication [9] (Table 2). The 5′ and 3′ ITRs were sequence-symmetrical and contained CRSs at homologous positions, matching a previously described goatpox virus (GTPV) CRS [5]. The incomplete base-pairing of poxvirus telomeres precludes the accurate representation of the terminal sequences of native genomes. Therefore, the hairpin sequences are shown in the ‘unfolded’ state in the annotated sequence. The schematic in Fig. 1 illustrates the differences between the annotated (Fig. 1a) and native (Fig. 1b) hairpin ends of LSDV Oman 2009. The lowest energy prediction of the structure of LSDV genome hairpin termini (Fig. 1c) shows the extra-helical loops and mismatched nucleotides typical of poxvirus genomes [39].

**Table 2.** CRS nucleotide sequence at the ITRs in the LSDV Oman 2009 genome assembly

Sequence	Position
TACTTTTTTTTTAGCCTATAAAT	1–23, 150,969–150,991
ATTTATAGGCTAAAAAAAAAGTA	101–123, 151,068–151,091

**Fig. 1.**
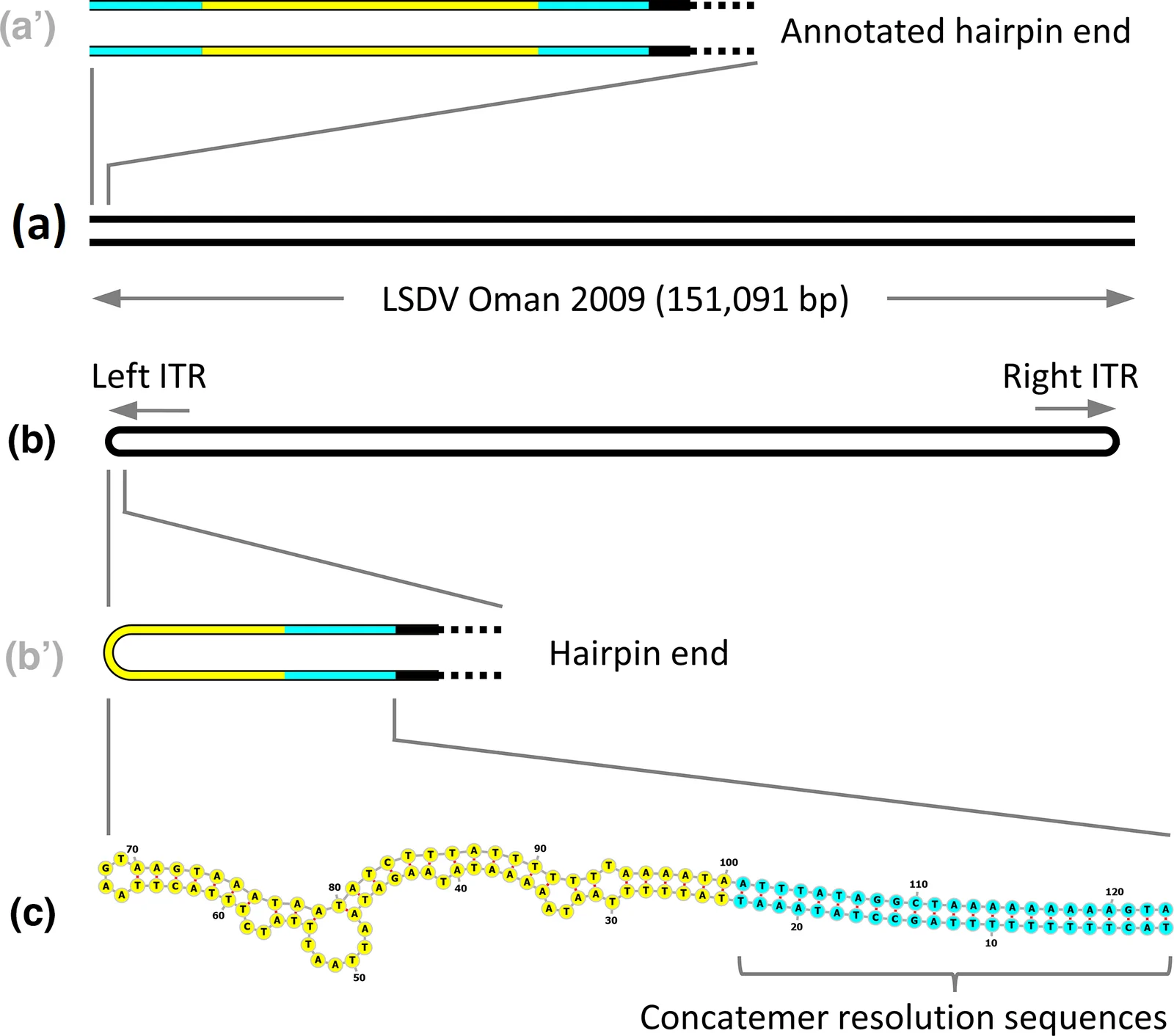
Map illustrating the structure of the LSDV Oman 2009 genome. Genome (**a**) represents the annotated T2T genome sequence of LSDV Oman 2009 as available on GenBank (accession number PV877838.1). Genome (**b**) represents the native LSDV genome showing the covalently linked ends. (**a’**) and (**b’**) show the enlarged left telomere to illustrate the organization of the annotated and native hairpin ends, respectively. Panel (**c**) shows the predicted structure of the left hairpin of LSDV Oman 2009 with the concatemer resolution site (1–23 :101–123) in cyan and incompletely paired hairpin sequence (24–100) in yellow.

To assess assembly quality, we visualized per-base read depth for both short- and long-read data across the genome (Fig. 1). Long-read depth was relatively uniform (Fig. 2d), with a sd of 66 (7% of the median depth of 962), compared with greater variability in short-read depth (sd 2,433; 23% of the median depth of 10,379) (Fig. 2a). Examination of the genome annotation additionally identified two truncated genes, LSDV019 and LSDV026, which are also truncated in other wild-type LSDV isolates from clade 1.1 [40–42]. Gene fragments LSDV019a/b and LSDV026a/b were present as truncated coding sequences matching LSDV019 and LSDV026, respectively, in the Neethling isolate NI-2490 from clade 1.2 (AF325528.1), where the genes are full length. Importantly, all valid reads showed the truncated allele without evidence of minority sequence variation. This has also been the pattern in read libraries from other clade 1.2 LSDV sequences that also contain truncated LSDV019 and LSDV026 but were not examined in this study. LSDV019 is homologous to kelch-like virulence factors characterized in orthopoxviruses [43–45]. LSDV026 is also truncated in sheeppox virus (SPPV) and GTPV and is an ortholog of vaccinia virus F11, a virulence factor involved in regulation of virus spread [46].

**Fig. 2.**
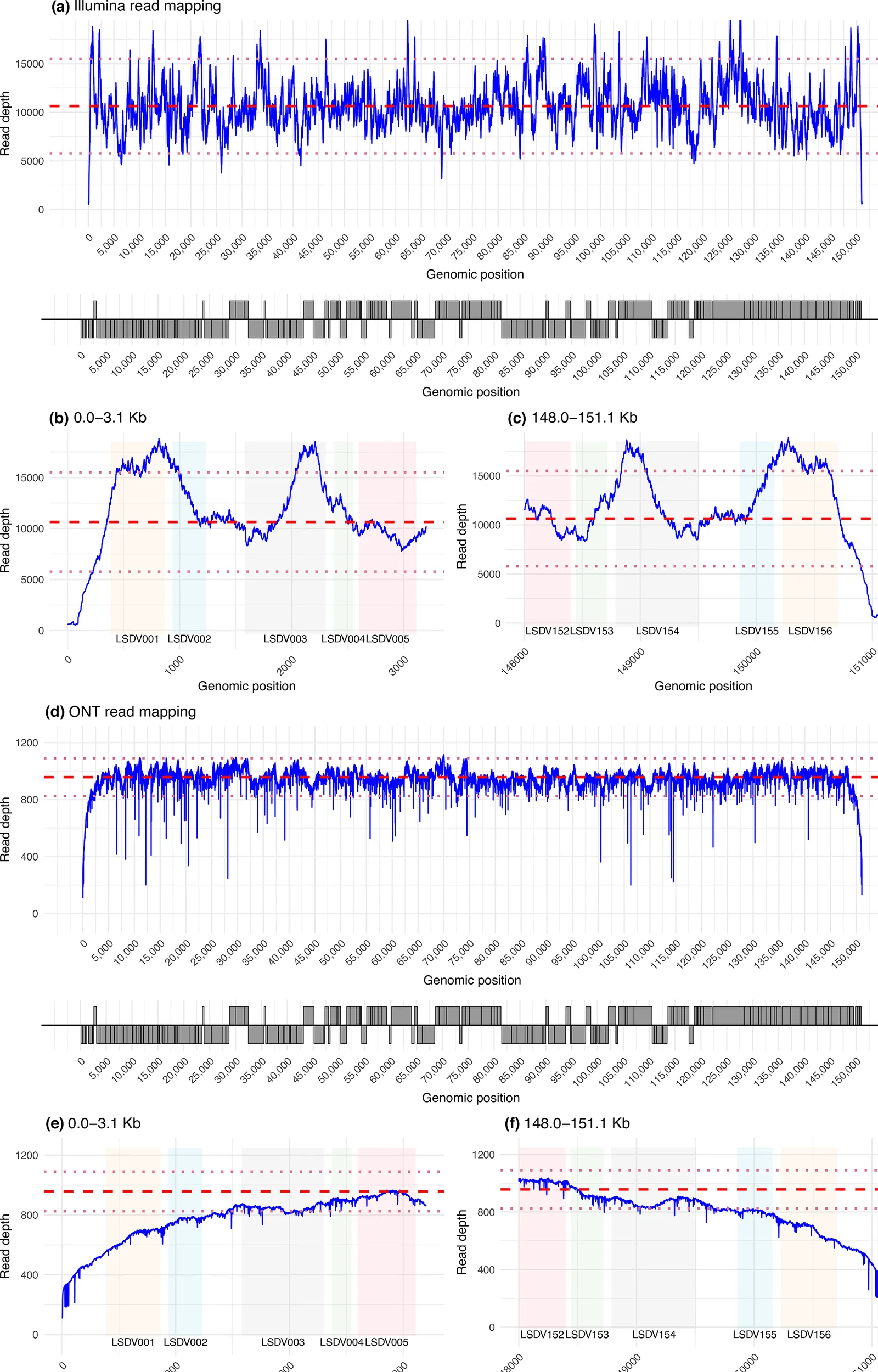
Per-base read depth across the LSDV Oman 2009 assembly for Illumina data (**a**) genome-wide, (**b**) at 0–3.1 kb and (**c**) at 148–151.1 kb, and equivalent ONT data within the same regions (**d**) genome-wide, (**e**) at 0–3.1 kb and (**f**) at 148–151.1 kb. The blue lines show read depth per site. The red dashed lines show genome-wide average read depth. The red dotted lines indicate twice the sd above and below the average. The small black boxes in (**e**) indicate the CRS. The ITRs are at 1–2,547 and 148,557–151,091 bp. The grey panels below (**a**) and (**d**) represent locations of annotated CDSs across the genome. Coloured shading in (**b**), (**c**), (**e**) and (**f**) demarcates LSDV001 (beige), LSDV002 (pale blue), LSDV003 (pale purple), LSDV004 (pale green), LSDV005 (pink), LSDV152 (pink), LSDV153 (pale green), LSDV154 (pale purple), LSDV155 (pale blue) and LSDV156 (beige).

Furthermore, we annotated a new ORF, tentatively named LSDV042.5, which is predicted to be an orthologue of vaccinia virus O3L. O3L encodes a 35-amino-acid protein that is a component of the EFC required for virus penetration into host cells and conserved across poxviruses of vertebrates [47, 48]. LSDV042.5 encodes a predicted polypeptide of 29 aa residues and therefore was not annotated in the first LSDV genome sequenced (AF325528.1) even when a sensitive cutoff (ORFs >30 aa residues) was applied [41]. An AlphaFold3 (AF3) model of LSDV042.5 revealed an alpha-helical protein predicted with overall high confidence (Fig. 3a, b). The structure of the 15-protein EFC of vaccinia virus was determined by cryogenic electron microscopy and revealed two O3 copies in the complex periphery [37]. The structural similarity with vaccinia virus O3 revealed by superimposing AF3 model on the EFC structure (Fig. 3c; root-mean-square deviation=1.019 Å) suggests that LSDV042.5 is likely to be functional. The fact that the O3L-like gene from SPPV (AY077832.1), which is 93% identical to LSDV042.5, can complement O3L deficiency in a recombinant vaccinia virus [47] corroborates the biological significance of LSDV042.5.

**Fig. 3.**
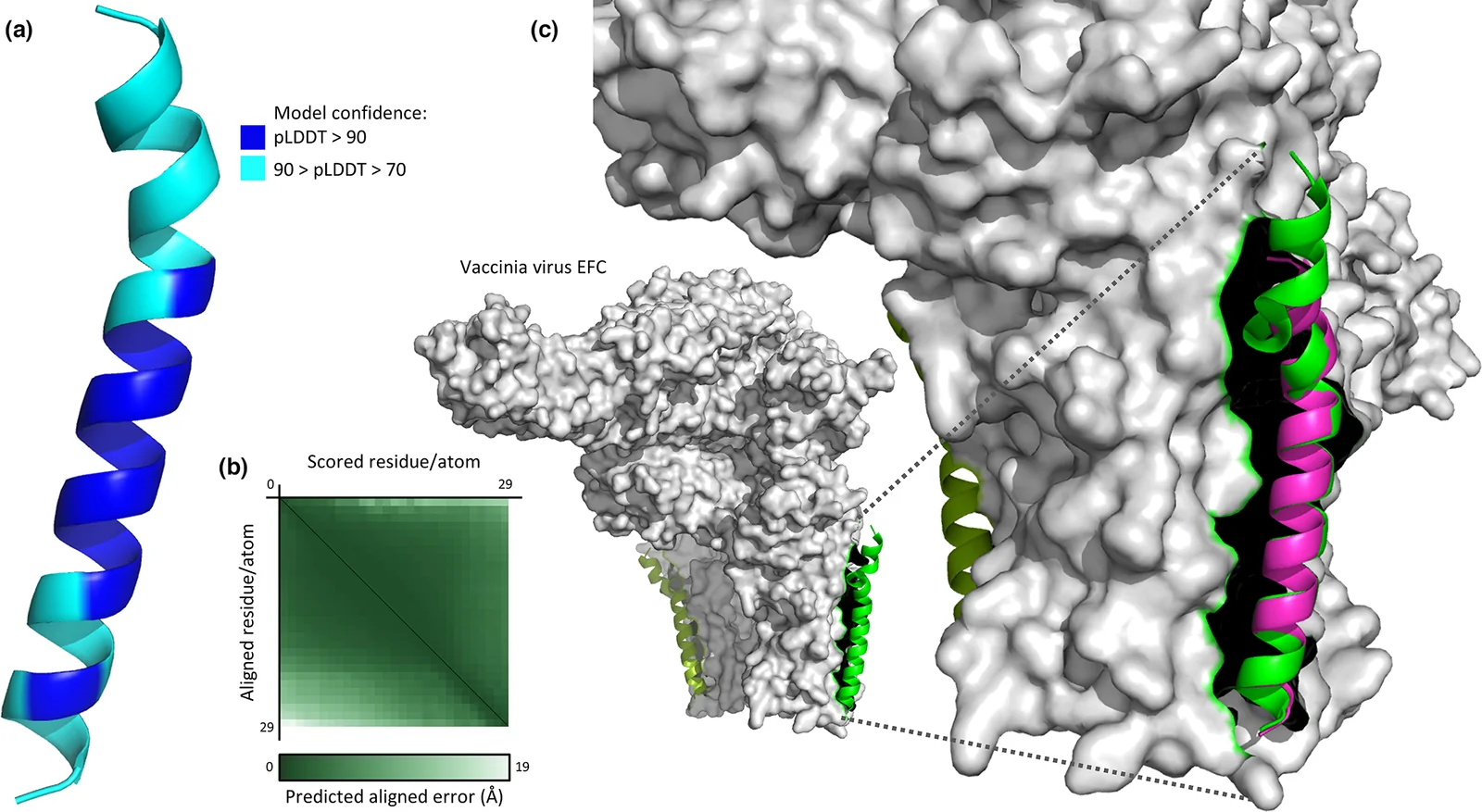
LSDV042.5 is a predicted component of the EFC. (**a**) AF3 model of LSDV042.5 protein with amino acid residues coloured according to the per-residue predicted local difference test (pLDDT). (**b**) PAE of LSDV042.5 model. (**c**) Superimposition of LSDV042.5 model on the structure of EFC from vaccinia virus determined by cryogenic electron microscopy (PDB 9UZO). The structure of the 15-subunit EFC is shown on the bottom left with the two O3 subunits displayed in shades of green. On the right, the zoom in highlights LSDV042.5 (magenta) superimposed on one of the O3 subunits (green).

The reference genome contained a 5′ ITR spanning positions 1–2,547 bp and a corresponding 3′ ITR spanning positions 148,557–151,091 bp, yielding ITR lengths of ~2.5 kb at each terminus. This annotation was supported by the genomic positions of conserved flanking genes, including LSDV005 (2,599–3,111 bp) at the 5′ end and an ankyrin repeat protein (LSDV152; 146,929–148,398) at the 3′ end. Analysis of read depth revealed abrupt changes in short-read coverage at ~1.2, 3.3, 5.8 and 6.4 kb from the 5′ end (Fig. 2b), with corresponding features observed at the 3′ end of the genome (Fig. 2c). These coverage transitions were present when short reads, but not long reads, were mapped to the reference (Fig. 2e, f). Median short-read depth across the 5′ and 3′ ITRs (12,580 and 12,592, respectively) exceeded the genome-wide median short-read depth of 10,379, indicating overrepresentation of these regions due to their repetitive structure. This suggests that short reads alone overestimate ITR length by 22%, whereas long reads spanned the repetitive regions and enabled accurate estimation of ITR structure and length.

Alignment of the Oman 2009 hybrid assembly with the PacBio-based genome assembly PX492334 [11] revealed only 12 mismatches across the full genome length. PX492334 genome is derived from a different LSDV isolate (Israel/2012), and therefore, these changes may reflect the underlying genetic backgrounds of each isolate [2] rather than ascertainment differences. Notwithstanding, this high level of concordance indicates that both ONT and PacBio sequencing approaches can generate highly accurate assemblies for LSDV. Comparison of the terminal regions showed that the first 67 bp of the Oman 2009 assembly, including the first CRS at 1–23 bp (Table 2), was not present in the 5′ ITR of PX492334, where only a partial 11 bp match was observed; an identical pattern was detected at the 3′ end. Importantly, the presence of this additional sequence symmetrically at both genome termini in the Oman 2009 assembly supports its authenticity and suggests that the hybrid ONT-Illumina approach resolved an additional 56 bp at each end of the genome relative to the PacBio-only assembly.

## Supplementary material

10.1099/jgv.0.002348 Supplementary Material 1.

10.1099/jgv.0.002348 Supplementary Material 2.

## Abbreviations

AF3: AlphaFold3
BQ: base quality
CaPV: capripoxvirus
CRS: concatemer resolution sequence
EFC: entry fusion complex
ITR: inverted terminal repeat
LSDV: lumpy skin disease virus
PAE: predicted aligned error
SNP: single nucleotide polymorphism
T2T: telomere-to-telomere
WOAH: World Organization for Animal Health
