# Supplementary material for: A high-quality telomere-to-telomere lumpy skin disease virus genome assembly
Source: J Gen Virol. 2026 Oct 14;107(10):002348. doi: 10.1099/jgv.0.002348 (PMC13668194; doi:10.1099/jgv.0.002348)
Supplement: Supplementary file 1 [file jgv-107-02348-s001.pdf]

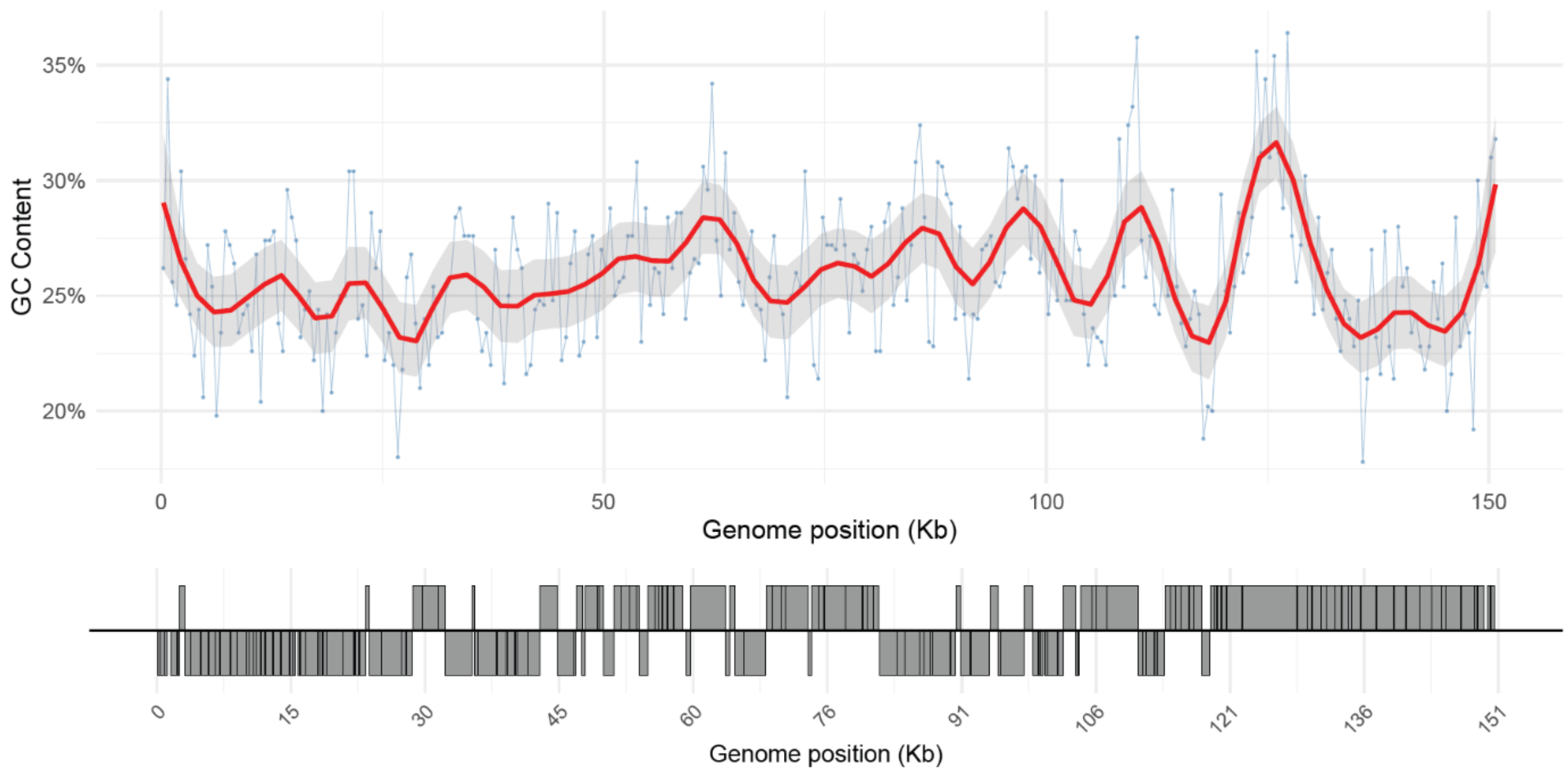

Figure S1. The genome-wide variation in GC content in the LSDV Oman 2009 hybrid genome assembly. The red line shows the smooth pattern in 500 bp bins and the grey area shows the 95% confidence interval. The grey panel represents the locations of the annotated CDSs across the genome. The y-axis has been cut.

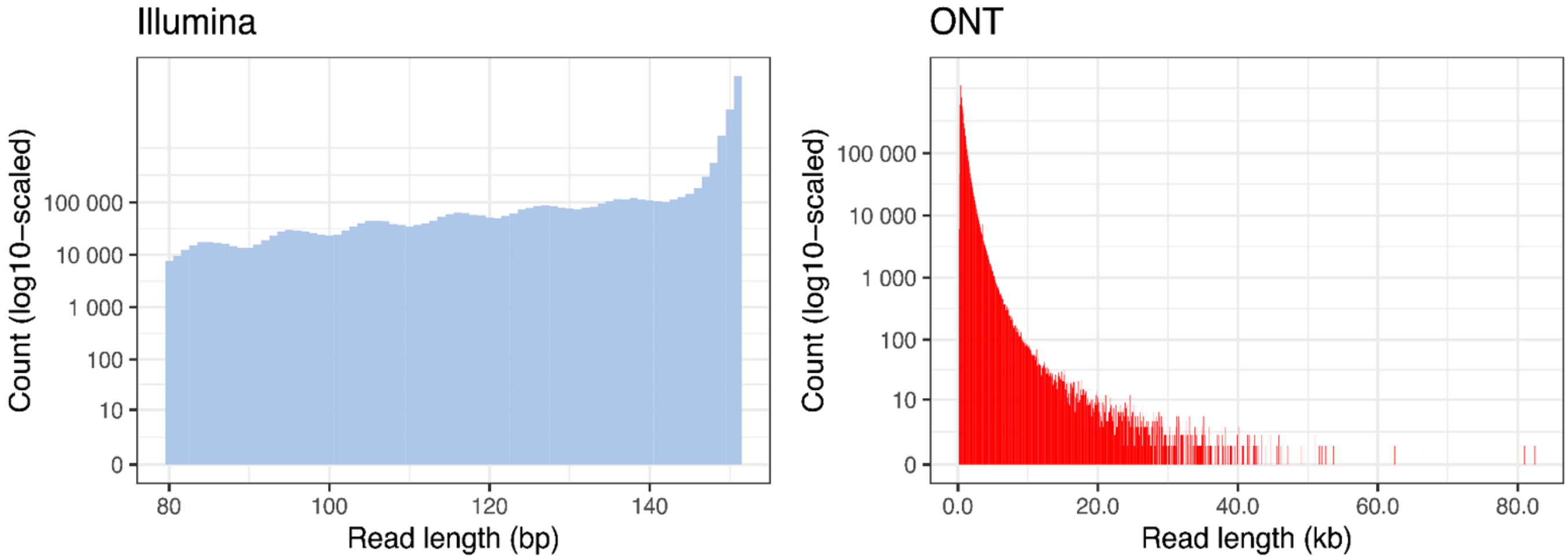

Figure S2. Histograms of the read length distributions for Illumina and ONT read libraries for reads with lengths > 80 bp. The Illumina reads lengths post-QC ranges from 80 to 151 bp; for ONT reads, this range was 25-82,438 bp.
